# Shall We Have a Quarantine War?

**Published:** 1899-03

**Authors:** 


### Abstracts and Selections.

### Shall We Have a Quarantine War?

The Mississippi Board of Health at a recent meeting seriously debated the advisability of placing New Orleans in the same category with Havana, Vera Cruz and Rio Janiero, as a suspicious and infected place against which quarantine should be declared in the spring without waiting for formal notice of the actual appearance of yellow fever.

This radical policy had many supporters throughout Mississippi, especially among the newspapers, and even in some of the country districts of Louisiana. It would at least have been a straight-out declaration of war. It was deemed advisable, however, not to take so radical a step at once, but to warn New Orleans that unless it consented to the terms proposed by Mississippi, a quarantine war would be declared. The demand of the Mississippi Board is contained in the proposition submitted to Louisiana and New Orleans a few days ago. It declares that "New Orleans is a menace to the health of the people of Mississippi on account of the lax management of epidemic contagious diseases by the health authorities," and that "Dr. Edward Souchon, President of the Louisiana State Board of Health, and Dr. Quitman Kohnke, President of the New Orleans Municipal Board of Health, are in favor of concealing yellow fever when it appears in New Orleans, Dr. Kohnke denouncing the law of the State which requires physicians to report cases of yellow fever, and refusing to inform the country when these cases have been reported to him by physicians of his city." These officers are charged not only with suppressing the truth, but also with violation of an agreement made by the Health Boards of the several Southern States at Atlanta, in 1898, being influenced to do so by the commercial exchanges of New Orleans; in other words, it is asserted that the truth about yellow fever was suppressed at New Orleans for fear that it might disturb the business of the city, and that the disease was allowed to spread secretly from that city and be scattered throughout the Southwest. The Mississippi Board winds up its charges with the specific declaration that the disease was introduced in this country last year by way of New Orleans and scattered through the Southwest from that city. It accordingly announces that unless its demands is complied with it will declare a quarantine against New Orleans in April or May, shutting that city completely out of Mississippi. If Mississippi should do this the effect would be very serious to New Orleans, as a large part of the travel and traffic to and from New Orleans passes through that State.

The following summary of the existing conditions, taken from the *New York Sun,* will be read with much interest:

"The demand made as a condition of peace is that a representative of the Mississippi State Board of Health shall be officially stationed at New Orleans, very much as the United States send Ambassadors to England and other countries to protect American interests abroad. This foreign representative must have plenary powers far beyond what an Ambassador possesses. He must be permitted to make a house-to-house inspection of any part of New Orleans at any time; he must be permitted to see all cases of illness considered to be suspicious; to visit and inspect all the city hospitals at any time and without securing any special permit.

"The conditions are onerous and are not likely to be accepted. The Galveston health authorities refused to agree to similar conditions last year when requested to do so by the Louisiana State Board. The Louisiana and New Orleans authorities rejected these propositions a year ago when Mississippi presented them. It is not possible, they say, to allow outside health authorities to invade a private residence at any time. As for seeing the sick, the Louisiana health authorities themselves may not do so if the family objects. If every woman or child who became ill had to submit to a clinical inspection by health officers, not from Mississippi alone, but also from Alabama, Texas, and possibly other States—for all of them ought to enjoy exactly the same privileges as Mississippi—there will be small hope for the patient. No attending physician, they say, would consent to have his patient thus persecuted.

"The Louisiana and New Orleans Boards of Health have not yet replied to the demand, but it is quite certain that they will reject it. Even if they consented, the people of New Orleans would not permit the proposed system to be put into operation. They are very ugly on this subject, and the Olliphant Board of Health, ousted after the epidemic of 1897, was driven out of office largely because it interfered with the people in their houses. If the boards reply to the Mississippi demand it will be to deny all the charges made against Louisiana and New Orleans, and to assert that yellow fever appeared first in Mississippi, at McHenry, as early as May, and that it was well circulated throughout Mississippi before it made its appearance in New Orleans, having been imported (as a leading member of the Mississippi Board himself showed after investigation) by a bridge gang which came from Santiago after serving there with General Shafter.

"The only reply so far made to the Mississippi Board is a libel suit, which may be considered one of the initial skirmishes of the coming quarantine war. The charges made against Louisiana and New Orleans were based on a long story of the last epidemic by a Dr. McKowen or McKewan—strangely enough published in the Baton Rouge *Advocate,* the official organ of the State of Louisiana, which thus attacks a Louisiana State official. Dr. Kohnke declares the article libelous, and has sued Dr McKowen or McKewan for $25,000 damages. Very little is known about the latter, who has been in New Orleans only a few months. His charges in the article certainly appear exaggerated, for he asserts that more than 20,000 cases of yellow fever occurred in New Orleans last summer. Dr. Kohnke thinks it strange that under these circumstances Dr. McKewan did not see or report a case to him, as the law required.

"This is the situation so far. As for the threatened war, it will not be a simple fight of two bodies, but a dozen or more will be engaged. The Mississippi Board of Health is pledged to declare quarantine against New Orleans and Louisiana if its terms are rejected. It is almost certain that Texas and Alabama will do the same; indeed, the Texas Health Officer has just obtained an opinion from the Attorney-General of the State, assuring him of his power to declare State quarantines. Last year there was considerable friction between the State Boards of Alabama and Mississippi. As the former State altogether escaped the fever, while the disease prevailed at more than thirty points in Mississippi, it is likely to quarantine against Mississippi early in the action, and Texas will undoubtedly include Mississippi with Louisiana in any quarantine it may declare; indeed, the chances are that the Lone Star State will stop all transit from the East, admitting passengers and freight only through Texarkana and the Indian Territory. The Mississippi State Board is in bad odor in many parts of the State, and, with the panicky feeling prevailing there, an exchange of quarantines among the several towns and counties is certain. The coast counties of Mississippi suffered so from quarantine last year, which shut up their factories, suspended their business and cut down the value of their real estate one-half, that they gave notice at a recent meeting of business men that they preferred the yellow fever to quarantine, and in consequence would secede, so far as health matters are concerned, from Mississippi if the State Board of Health quarantined against New Orleans. They threaten a suit against their own State Board in case it goes into the quarantine business ill-advisedly and unnecessarily, citing in support of such a suit the opinion of Chief Justice Woods, of Mississippi, given in the case of the town of Kosciusko, against Stomberg, that a quarantine against a town in the absence of an epidemic was an unreasonable restraint upon trade and therefore void, and no one can be prosecuted for violating it.

"If the Mississippi coast counties are antagonizing the State Board of Health of Mississippi, the towns and parishes of western and northwestern Louisiana are similarly threatening their own State Board. The people of Crowley, Lake Charles and other towns on the Southern Pacific Railroad which are deeply interested in keeping the railroad open to Texas are disposed to side with Texas in the matter of quarantine, and on the first occasion offered will probably quarantine against New Orleans and all points to the East if Texas will consent to keep up communication with them and to agree that all trains shall be stopped at Franklin or New Iberia, La., instead of at the State line.

"When it is further said that the Louisiana State Board of Health, which has its headquarters at New Orleans, and the New Orleans Municipal Board of Health are at odds and may become involved in a serious quarrel any day, some idea may be had of the possibilities of the threatened quarantine war. It will be a case of a foreign war, supplemented by half a dozen civil wars at the same time. And this conflict may be precipitated without a single case of fever occurring in the South. The demand made by Mississippi on New Orleans is accompanied with the threat of quarantine unless it is granted. Mississippi will not wait for a case to be reported, but will act on suspicion as a means of preventing the introduction of yellow fever.

"Already business is beginning to feel the depression that the threat of conflict causes. Dr. Souchon, President of the Louisiana Board of Health, tried to mitigate the evil by calling a quarantine conference this week to amend the Atlanta agreement. That agreement, made in April, 1898, provided for carrying on trade even during periods of epidemics or quarantines. It worked well last year, but several defects were discovered in it, and the New Orleans quarantine conference was for the purpose of remedying them. But the New Orleans conference was not well attended, nor was it able to accomplish much. At best, trade carried on under the Atlanta agreement is slow, tedious and expensive. It is not quite so bad as the condition prevailing in 1897, when the people in many southern towns suffered for lack of food, medicine and the necessaries; but it is bad enough, and means stagnation, loss and suffering."

The conditions outlined above are the natural result of the failure of Congress to enact any general quarantine law, and these deplorable conditions will continue to obtain until Congress can be brought to enact some efficient and equitable quarantine legislation. The States at interest would much better submit to the sacrifice of some of their own powers to the Federal government than to suffer the constant recurrence of the panic and loss of 1898.—*Gaillard's Medical Journal.*

